# Surgical Resection for Colorectal Liver Metastasis in Elderly Patients Aged ≥ 80: A Retrospective Nationwide Cohort Survey in Japan With Propensity Score Matching

**DOI:** 10.1002/ags3.70213

**Published:** 2026-03-10

**Authors:** Kiichi Sugimoto, Akio Saiura, Shuko Nojiri, Hirotoshi Kobayashi, Goro Honda, Kazushige Kawai, Kiyoshi Hasegawa, Keiichi Takahashi

**Affiliations:** ^1^ Department of Coloproctological Surgery Juntendo University Faculty of Medicine Tokyo Japan; ^2^ Department of Hepatobiliary‐Pancreatic Surgery Juntendo University Faculty of Medicine Tokyo Japan; ^3^ Medical Technology Innovation Center Juntendo University Faculty of Medicine Tokyo Japan; ^4^ Department of Surgery Teikyo University Hospital, Mizonokuchi Tokyo Japan; ^5^ Department of Surgery, Institute of Gastroenterology Tokyo Women's Medical University Tokyo Japan; ^6^ Department of Colorectal Surgery Tokyo Metropolitan Cancer and Infectious Diseases Center Komagome Hospital Tokyo Japan; ^7^ Hepato‐Biliary‐Pancreatic Surgery Division, Department of Surgery, Graduate School of Medicine The University of Tokyo Tokyo Japan; ^8^ Department of Surgery Tokyo Metropolitan Health and Hospitals Corporation Ohkubo Hospital Tokyo Japan

**Keywords:** colorectal liver metastasis (CRLM), elderly patients, liver resection, propensity score matching, super‐aged society

## Abstract

**Background:**

As the aging of the global population increases, the number of elderly patients with colorectal liver metastasis (CRLM) is expected to increase. The aim of this study was to clarify the long‐term oncological outcomes and treatment of CRLM in elderly patients aged ≥ 80.

**Methods:**

Patients with CRLM were identified from the Japanese database of Liver Metastases Survey of Colorectal Cancer, collected from 145 centers. Two thousand four hundred nine patients with liver resection were eligible for analysis. This cohort was divided into two groups aged < 80 (group Y (younger); *n* = 2194) and ≥ 80 (group E (elderly); *n* = 215). We compared the long‐term outcomes after liver resection of patients with CRLM aged ≥ 80 and < 80 years after adjustment using propensity score matching (PSM), and we identified the reasons for differences in these outcomes.

**Results:**

After PSM, group E showed a comparable recurrence‐free survival to that in group Y (hazard ratio (HR): 1.20, *p* = 0.16). However, group E had significantly worse cancer‐specific survival (CSS) (HR: 1.77, *p* = 0.002) and overall survival (OS) (HR: 1.80, *p* = 0.0004). Treatment for recurrence was given at a significantly lower rate in group E (80/130 (61.5%) vs. 1148/1329 (86.4%), *p* < 0.0001).

**Conclusions:**

Surgical resection seems to be as effective in elderly patients aged ≥ 80 as in nonelderly patients. The significantly worse CSS and OS may have resulted from a lower rate of treatment for recurrence in elderly patients aged ≥ 80. Physicians should keep this finding in mind with regard to limitations of treatments for recurrence after initial liver resection in elderly patients aged ≥ 80 with CRLM.

## Graphical abstract

Surgical resection for CRLM seems to be as effective in elderly patients aged ≥ 80 as in nonelderly patients. However, elderly patients aged ≥ 80 had significantly worse cancer‐specific survival (CSS) and overall survival (OS). The significantly worse CSS and OS may have resulted from a lower rate of treatment for recurrence in elderly patients aged ≥ 80.

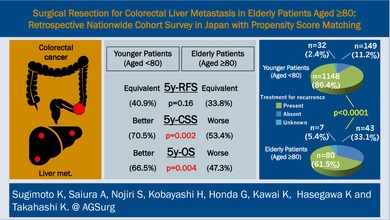

## 1 Introduction

Colorectal cancer (CRC) is a major cause of mortality worldwide and is increasingly common in Japan [1, 2]. However, liver resection is now indicated in more cases and several new systemic chemotherapy regimens have been introduced, with subsequent improvement of postoperative outcomes for cases with resectable colorectal liver metastases (CRLM) [3, 4]. In such cases, liver resection is currently viewed as the most effective treatment [5, 6, 7], but recurrence is still a concern, even after curative resection [8, 9, 10, 11]. Therefore, post‐resection treatments that are more effective and aggressive are required for patients with CRLM.

The world is aging and an increasing number of countries are becoming aged or super‐aged societies [12]. Japan has already become the first super‐aged society [12]. As aging of the global population increases, the number of elderly patients with CRLM is also expected to increase [13, 14]. The prevalence of frailty is high in elderly patients, and the postoperative morbidity and mortality of these patients is also likely to be high [15]. For these reasons, the proportion of elderly patients who are eligible for curative surgery for CRLM is low, and multidisciplinary treatments, including surgical approaches, for these patients need to be less aggressive [14, 16, 17]. To examine the usefulness and limitations of such treatments in elderly patients with CRLM, there is a need to compare treatment and long‐term outcomes in elderly and nonelderly patients. However, differences in patient backgrounds and tumor characteristics between these patient populations make such a study challenging.

To overcome this issue, propensity score matching (PSM) has been used to eliminate biases between these groups [14, 18]. However, these studies have included relatively small cohorts of less than 100 elderly patients, and the numerous surgical options available for CRLM leads to inconsistencies in decision‐making among surgeons when choosing a therapeutic strategy [19]. Therefore, a large cohort of elderly patients with CRLM is required to obtain conclusive results. Also, the “elderly” are not clearly defined as a homogeneous population [20], although in most recent studies, age 70 has been used as a cut‐off to define the elderly population when considering hepatectomy [21]. The rate of patients aged 70 or above undergoing surgical resection for CRLM has recently increased [20] and information is available on the subsequent short‐ and long‐term outcomes in this population [14, 20]. In contrast, there are only a few reports of similar outcomes in patients aged 80 or older with CRLM [22, 23].

The average life expectancy in Japan in 2022 has become 81.05 years for men and 87.09 years for women, representing the world's longest longevity [24]. Life expectancy of the global population has continued to increase and is now over 80 years in many countries [25], emphasizing the importance of assessment of treatment of CRLM in patients aged 80 or older. Therefore, in this study, we compared the long‐term outcomes after liver resection of patients with CRLM aged ≥ 80 and < 80 years after adjustment using PSM, and we identified the reasons for differences in these outcomes.

## 2 Methods

### 2.1 Patient Selection

The scheme of the study is shown in Figure S1. Patients with CRLM were identified from the database of the Joint Committee of Liver Metastases Survey of Colorectal Cancer, collected from 145 Japanese centers. This database contains prospective data on patients diagnosed with CRLM from 2013 to 2017, with survival data collected in 2018 and 2022 [26]. A total of 7828 patients with CRLM with available survival data were identified. Of these patients, 3476 without liver resection were excluded, along with patients with simultaneous extrahepatic metastasis (*n* = 518), incurable liver resection (*n* = 69) and missing data (*n* = 1356). This left 2409 patients who were eligible for analysis. This cohort was divided into two groups aged < 80 (group Y (younger); *n* = 2194, 91.1%) and ≥ 80 (group E (elderly); *n* = 215, 8.9%). Race/ethnicity data were not available due to the lack of the detailed information in this database. Synchronous metastases were defined as liver metastatic disease at presentation. The study was conducted in accordance with the Declaration of Helsinki, and was approved by the institutional review board (IRB) of Tokyo Metropolitan Cancer and Infectious Diseases Center Komagome Hospital (No. 1168). The requirement for formal informed consent was waived because of the retrospective design.

### 2.2 Preoperative and Postoperative Adjuvant Chemotherapy

Age and clinicopathological factors were taken into account in the choice of adjuvant chemotherapy regimen. The final decision on the regimen for each patient was reached after a discussion of the benefits and disadvantages between the physician and the patient.

### 2.3 Clinicopathological Analysis

Clinicopathological factors gender, American Society of Anesthesiologists (ASA) classification (≤ 2/≥ 3), location of primary tumor (colon/rectum), undifferentiated component in primary tumor (absent/present), depth of tumor invasion (pT/ypT1‐3/pT/ypT4), lymph node metastasis (pN/ypN 0/pN/ypN 1, 2), emergence time of CRLM (synchronous/metachronous), distribution of CRLM (unilobar/bilobar), number of CRLMs (≤ 3/≥ 4), maximum diameter of CRLM (< 50 mm/≥ 50 mm), liver resection procedure (anatomical/partial), surgical curability (R0/R1), postoperative complication of Clavien‐Dindo (C‐D) classification Grade 3 or higher after liver resection (absent/present), preoperative adjuvant chemotherapy for CRLM (absent/present), postoperative adjuvant chemotherapy for CRLM (absent/present), and survival were analyzed. Surgical curability was defined as: R0, no residual tumor; and R1, a microscopic residual tumor [27, 28].

### 2.4 Propensity Score Matching (PSM)

Differences in clinicopathological severity between groups Y and E were adjusted by PSM [29, 30]. The PS was estimated and the log odds of the probability for a patient were modeled with potential confounders of clinicopathological factors with *p* < 0.05 in univariate analyses between groups Y and E. Propensity model discrimination was evaluated using the c‐statistic. One‐on‐one pair PSM analysis using calipers < 0.01 was conducted in JMP Pro 18 (SAS Institute Inc., Cary, NC, USA). Standardized differences were used for analysis of matched outcomes. A standardized difference < 0.10 suggests adequate variable balance in PSM [31].

### 2.5 Statistical Analysis

Recurrence‐free survival (RFS: time from initial CRLM surgery to first recurrence), cancer‐specific survival (CSS: time from initial CRLM surgery to cancer‐related death), and overall survival (OS: time from initial CRLM surgery to all‐cause mortality) were determined using the Kaplan–Meier method, with significance determined by univariate analyses with a log‐rank test. Clinicopathological factors that differed significantly in univariate analysis were included as covariates in multivariate analysis using a Cox proportional‐hazard regression model that allowed calculation of a hazard ratio (HR) and confidence interval (CI). For multivariate analysis, a logistic regression model was used with the odds ratio (OR) and CI as a measure of association of clinicopathological factors. Discrete and nominal variables were compared by Fisher exact test and continuous variables by Mann–Whitney *U*‐test. JMP Pro 18 was used for all analyses, with *p* < 0.05 taken to indicate a significant difference. Data are reported as a median with the minimum—maximum range in parentheses.

## 3 Results

### 3.1 Clinicopathological Factors

Univariate analysis indicated that ASA classification at resection of CRLM, emergence time of CRLM, distribution of CRLM, number of CRLMs, preoperative adjuvant chemotherapy for CRLM and postoperative adjuvant chemotherapy for CRLM differed significantly between groups Y and E. Patients in group E more frequently had an ASA classification at resection of CRLM ≥ 3 (*p* = 0.006) and less frequently had synchronous CRLM (*p* < 0.0001), bilobar CRLM (*p* < 0.0001), number of CRLMs (≥ 4) (*p* < 0.0001), preoperative adjuvant chemotherapy for CRLM (*p* < 0.0001) and postoperative adjuvant chemotherapy for CRLM (*p* < 0.0001) (Table 1). There were no other significant differences in clinicopathological factors between the two groups.

**TABLE 1:** Clinicopathological factors in the groups Y and E before propensity score matching.

Clinicopathological factors	Variables	Before matching		*p*
		Group Y *n* = 2194 (%)	Group E *n* = 215 (%)	
Gender	Male, Female	1374 (62.6%) 820 (37.4%)	126 (58.6%) 89 (41.4%)	0.27
ASA classification at resection of CRLM^a^	≤ 2, ≥ 3	2113 (96.3%) 81 (3.7%)	198 (92.1%) 17 (7.9%)	0.006
Locations of primary tumor^b^	Colon, Rectum	1679 (76.5%) 515 (23.5%)	173 (80.5%) 42 (19.5%)	0.20
Undifferentiated component in primary tumor	Absent, Present	1999 (91.1%) 195 (8.9%)	202 (94.0%) 13 (6.1%)	0.20
Depth of tumor invasion	pT/ypT 1–3, pT/ypT 4	1581 (72.1%) 613 (27.9%)	152 (70.7%) 63 (29.3%)	0.69
Lymph node metastasis	pN/ypN 0, pN/ypN 1, 2	788 (35.9%) 1406 (64.1%)	89 (41.4%) 126 (58.6%)	0.12
Emergence time of CRLM	Synchronous, Metachronous	1213 (55.3%) 981 (44.7%)	86 (40.0%) 129 (60.0%)	< 0.0001
Distribution of CRLM	Unilobar, Bilobar	1448 (66.0%) 746 (34.0%)	180 (83.7%) 35 (16.3%)	< 0.0001
Number of CRLM	≤ 3, ≥ 4	1727 (78.7%) 467 (21.3%)	194 (90.2%) 21 (9.8%)	< 0.0001
Maximum diameter of CRLM	< 50 mm, ≥ 50 mm	1864 (85.0%) 330 (15.0%)	188 (87.4%) 27 (12.6%)	0.37
Liver resection procedure^c^	Anatomical, Partial	771 (38.8%) 1217 (61.2%)	85 (42.5%) 115 (57.5%)	0.32
Surgical curability of CRLM	R0, R1	2081 (94.9%) 113 (5.2%)	200 (93.0%) 15 (7.0%)	0.26
Postoperative complication after liver resection ≥ Grade 3^d^	Absent, Present	1856 (91.5%) 173 (8.5%)	180 (89.1%) 22 (10.9%)	0.24
Preoperative adjuvant chemotherapy for CRLM	Absent, Present	1732 (78.9%) 462 (21.1%)	193 (89.8%) 22 (10.2%)	< 0.0001
Postoperative adjuvant chemotherapy for CRLM	Absent, Present	991 (45.2%) 1203 (54.8%)	173 (80.5%) 42 (19.5%)	< 0.0001

^a^ American Society of Anesthesiologists classification.

^b^ Multiple cancers were included. Cases involving rectal lesions were classified as ‘rectum’.

^c^ Data were missing in 221 patients.

^d^ Clavien‐Dindo classification, data were missing in 178 patients.

### 3.2 Prognostic Factors for RFS, CSS, and OS in Patients Aged < 80 Years

The results of univariate and multivariate analyses in group Y are shown in Table 2. All multivariate analyses used factors with significant differences in univariate analyses as covariates. In multivariate analysis for RFS, location of primary tumor (rectum), depth of tumor invasion (pT/ypT4), lymph node metastasis (pN/ypN1, 2), emergence time of CRLM (synchronous), distribution of CRLM (bilobar), number of CRLMs (≥ 4) and surgical curability (R1) were significant independent prognostic factors for RFS (Table 2).

**TABLE 2:** Prognostic factors for RFS, CSS and OS in patients aged < 80 years (group Y).

Clinicopathological factors	Variables (*n*)	Reference (*n*)	RFS			CSS			OS		
			Univariate	Multivariate		Univariate	Multivariate		Univariate	Multivariate	
			*p*	HR (95% CI)	*p*	*p*	HR (95% CI)	*p*	*p*	HR (95% CI)	*p*
Gender	Male (*n* = 1374)	Female (*n* = 820)	0.95	—	—	0.27	—	—	0.90	—	—
ASA classification at resection of CRLM^a^	≥ 3 (*n* = 81)	≤ 2 (*n* = 2113)	0.21	—	—	0.33	—	—	0.001	2.09 (1.44–3.03)	< 0.0001
Locations of primary tumor^b^	Rectum (*n* = 515)	Colon (*n* = 1679)	0.04	1.30 (1.13–1.50)	0.0002	0.04	1.50 (1.23–1.84)	< 0.0001	0.007	1.53 (1.27–1.84)	< 0.0001
Undifferentiated component in primary tumor	Present (*n* = 195)	Absent (*n* = 1999)	0.08	—	—	0.005	1.21 (0.90–1.61)	0.20	0.005	1.21 (0.92–1.58)	0.17
Depth of tumor invasion	pT/ypT 4 (*n* = 613)	pT/ypT 1–3 (*n* = 1581)	< 0.0001	1.47 (1.29–1.68)	< 0.0001	< 0.0001	1.44 (1.19–1.74)	0.0002	< 0.001	1.41 (1.18–1.68)	0.0002
Lymph node metastasis	pN/ypN 1, 2 (*n* = 1406)	pN/ypN 0 (*n* = 788)	< 0.0001	1.59 (1.39–1.82)	< 0.0001	< 0.0001	2.23 (1.79–2.77)	< 0.0001	< 0.0001	1.83 (1.52–2.22)	< 0.0001
Emergence time of CRLM	Synchronous (*n* = 1213)	Metachronous (*n* = 981)	< 0.0001	1.35 (1.18–1.55)	< 0.0001	< 0.0001	1.14 (0.93–1.40)	0.22	< 0.0001	1.12 (0.94–1.35)	0.21
Distribution of CRLM	Bilobar (*n* = 746)	Unilobar (*n* = 1448)	< 0.0001	1.41 (1.21–1.63)	< 0.0001	< 0.0001	1.46 (1.18–1.80)	0.0006	< 0.0001	1.40 (1.15–1.70)	0.0008
Number of CRLM	≥ 4 (*n* = 467)	≤ 3 (*n* = 1727)	< 0.0001	1.33 (1.13–1.57)	0.0006	< 0.0001	1.38 (1.10–1.74)	0.006	< 0.0001	1.33 (1.07–1.65)	0.01
Maximum diameter of CRLM	≥ 50 mm (*n* = 330)	< 50 mm (*n* = 1864)	< 0.0001	1.18 (0.99–1.40)	0.06	< 0.0001	1.36 (1.07–1.74)	0.01	< 0.0001	1.23 (0.98–1.54)	0.08
Liver resection procedure^c^	Anatomical (*n* = 771)	Partial (*n* = 1217)	0.01	1.08 (0.95–1.23)	0.22	0.02	1.12 (0.92–1.35)	0.26	0.002	1.21 (1.01–1.43)	0.03
Surgical curability of CRLM	R1 (*n* = 113)	R0 (*n* = 2081)	0.001	1.30 (1.02–1.66)	0.04	0.002	1.28 (0.92–1.79)	0.15	0.002	1.28 (0.93–1.75)	0.13
Postoperative complication after liver resection ≥ Grade 3^d^	Present (*n* = 173)	Absent (*n* = 1856)	0.005	1.07 (0.87–1.33)	0.52	0.048	1.18 (0.87–1.60)	0.27	< 0.0001	1.36 (1.04–1.78)	0.02
Preoperative adjuvant chemotherapy for CRLM	Present (*n* = 462)	Absent (*n* = 1732)	< 0.0001	1.11 (0.96–1.28)	0.15	0.03	1.06 (0.86–1.30)	0.61	0.09	—	—
Postoperative adjuvant chemotherapy for CRLM	Present (*n* = 1203)	Absent (*n* = 991)	0.56	—	—	0.009	1.21 (1.01–1.45)	0.04	0.17	—	—

^a^ American Society of Anesthesiologists classification.

^b^ Multiple cancers were included. Cases involving rectal lesions were classified as ‘rectum’.

^c^ Data were missing in 206 patients.

^d^ Clavien‐Dindo classification, data were missing in 165 patients.

In multivariate analysis for CSS, location of primary tumor (rectum), depth of tumor invasion (pT/ypT4), lymph node metastasis (pN/ypN1, 2), distribution of CRLM (bilobar), number of CRLMs (≥ 4), maximum diameter of CRLM (≥ 50 mm) and postoperative adjuvant chemotherapy for CRLM (present) were significant independent prognostic factors for CSS (Table 2).

In multivariate analysis for OS, ASA classification at resection of CRLM (≥ 3), location of primary tumor (rectum), depth of tumor invasion (pT/ypT4), lymph node metastasis (pN/ypN1, 2), distribution of CRLM (bilobar), number of CRLMs (≥ 4), liver resection procedure (anatomical) and postoperative complication after liver resection (C‐D classification ≥ Grade 3) were found to be significant independent prognostic factors for OS (Table 2).

### 3.3 Prognostic Factors for RFS, CSS, OS in Patients Aged ≥ 80 Years

The results of univariate and multivariate analyses in group E are shown in Table 3. As above, all multivariate analyses used factors with significant differences in univariate analyses as covariates. In multivariate analysis for RFS, depth of tumor invasion (pT/ypT4), lymph node metastasis (pN/ypN1, 2), emergence time of CRLM (synchronous), distribution of CRLM (bilobar) and maximum diameter of CRLM (≥ 50 mm) were significant independent prognostic factors for RFS (Table 3).

**TABLE 3:** Prognostic factors for RFS, CSS and OS in patients aged ≥ 80 years (group E).

Clinicopathological factors	Variables (*n*)	Reference (*n*)	RFS			CSS			OS		
			Univariate	Multivariate		Univariate	Multivariate		Univariate	Multivariate	
			*p*	HR (95% CI)	*p*	*p*	HR (95% CI)	*p*	*p*	HR (95% CI)	*p*
Gender	Male (*n* = 126)	Female (*n* = 89)	0.97	—	—	0.82	—	—	0.95	—	—
ASA classification at resection of CRLM^a^	≥ 3 (*n* = 17)	≤ 2 (*n* = 198)	0.74	—	—	0.88	—	—	0.62	—	—
Locations of primary tumor^b^	Rectum (*n* = 42)	Colon (*n* = 173)	0.45	—	—	0.65	—	—	0.86	—	—
Undifferentiated component in primary tumor	Present (*n* = 13)	Absent (*n* = 202)	0.28	—	—	0.72	—	—	0.66	—	—
Depth of tumor invasion	pT/ypT 4 (*n* = 63)	pT/ypT 1–3 (*n* = 152)	0.001	1.99 (1.36–2.90)	0.0004	0.0004	2.41 (1.48–3.93)	0.0004	0.005	2.50 (1.54–4.05)	0.0002
Lymph node metastasis	pN/ypN 1, 2 (*n* = 126)	pN/ypN 0 (*n* = 89)	0.0002	2.08 (1.42–3.06)	0.0002	0.003	1.86 (1.11–3.13)	0.02	0.02	1.94 (1.16–3.25)	0.01
Emergence time of CRLM	Synchronous (*n* = 86)	Metachronous (*n* = 129)	< 0.0001	1.72 (1.16–2.53)	0.007	< 0.0001	2.45 (1.46–4.13)	0.0007	< 0.0001	2.53 (1.51–4.25)	0.0004
Distribution of CRLM	Bilobar (*n* = 35)	Unilobar (*n* = 180)	0.001	1.70 (1.05–2.75)	0.03	< 0.0001	2.21 (1.20–4.06)	0.01	< 0.0001	2.08 (1.14–3.77)	0.02
Number of CRLM	≥ 4 (*n* = 21)	≤ 3 (*n* = 194)	0.001	1.09 (0.59–2.02)	0.77	0.002	0.55 (0.25–1.20)	0.13	0.02	0.52 (0.24–1.13)	0.10
Maximum diameter of CRLM	≥ 50 mm (*n* = 27)	< 50 mm (*n* = 188)	< 0.0001	1.82 (1.11–3.00)	0.02	0.0004	1.70 (0.90–3.22)	0.10	0.0001	1.71 (0.90–3.23)	0.10
Liver resection procedure^c^	Anatomical (*n* = 85)	Partial (*n* = 115)	0.55	—	—	0.99	—	—	0.64	—	—
Surgical curability of CRLM	R1 (*n* = 15)	R0 (*n* = 200)	0.07	—	—	0.049	1.78 (0.82–3.88)	0.14	0.15	—	—
Postoperative complication after liver resection ≥ Grade 3^d^	Present (*n* = 22)	Absent (*n* = 180)	0.87	—	—	0.09	—	—	0.07	—	—
Preoperative adjuvant chemotherapy for CRLM	Present (*n* = 22)	Absent (*n* = 193)	0.06	—	—	< 0.0001	2.66 (1.40–5.09)	0.003	< 0.0001	2.71 (1.42–5.19)	0.003
Postoperative adjuvant chemotherapy for CRLM	Present (*n* = 42)	Absent (*n* = 173)	0.01	1.35 (0.88–2.08)	0.17	0.79	—	—	0.68	—	—

^a^ American Society of Anesthesiologists classification.

^b^ Multiple cancers were included. Cases involving rectal lesions were classified as ‘rectum’.

^c^ Data were missing in 15 patients.

^d^ Clavien‐Dindo classification, data were missing in 13 patients.

In multivariate analysis for CSS, depth of tumor invasion (pT/ypT4), lymph node metastasis (pN/ypN1, 2), emergence time of CRLM (synchronous), distribution of CRLM (bilobar) and preoperative adjuvant chemotherapy for CRLM (present) were significant independent prognostic factors for CSS (Table 3).

In multivariate analysis for OS, depth of tumor invasion (pT/ypT4), lymph node metastasis (pN/ypN1, 2), emergence time of CRLM (synchronous), distribution of CRLM (bilobar) and preoperative adjuvant chemotherapy for CRLM (present) were significant independent prognostic factors for OS (Table 3).

### 3.4 RFS, CSS and OS in the Whole Cohort

**FIGURE 1:**
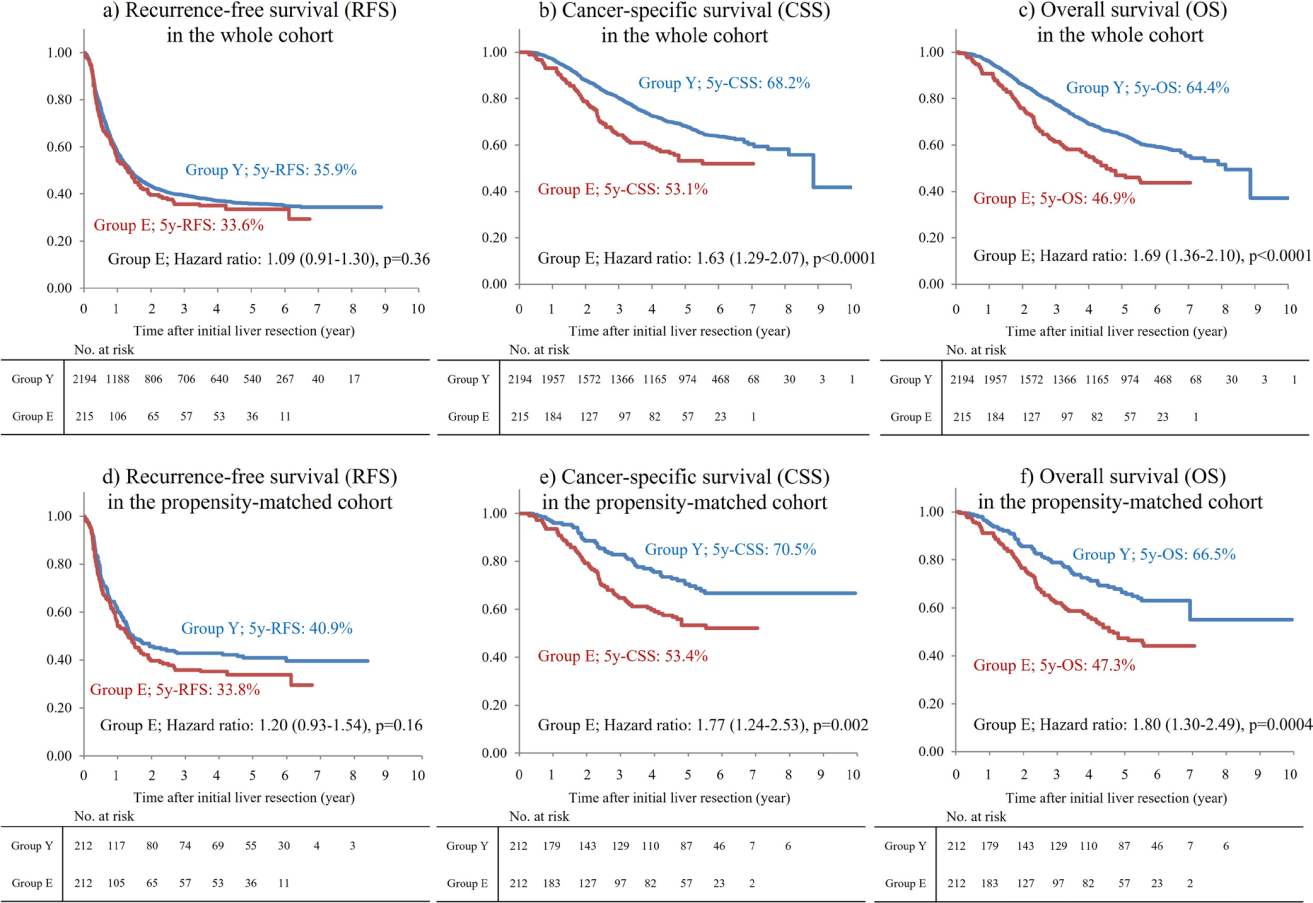
RFS, CSS and OS in the whole and the propensity‐matched cohorts Groups Y and E had no significant difference in RFS (Figure 1a), but group E had significantly worse CSS (Figure 1b) and OS (Figure 1c). In the PSM cohort, group E showed a comparable RFS to that in group Y (Figure 1d), and had significantly worse CSS (Figure 1e) and OS (Figure 1f).

Groups Y and E had no significant difference in RFS (5‐year RFS: group Y: 35.9%, group E: 33.6%) (HR: 1.09, 95% CI: 0.91–1.30, *p* = 0.36) (Figure 1a), but group E had significantly worse CSS (5‐year CSS: group Y: 68.2%, group E: 53.1%) (HR: 1.63, 95% CI: 1.29–2.07, *p* < 0.0001) (Figure 1b) and OS (5‐year OS: group Y: 64.4%, group E: 46.9%) (HR: 1.69, 95% CI: 1.36–2.10, *p* < 0.0001) (Figure 1c).

### 3.5 PSM

The PS was estimated using potential confounders that were clinicopathological factors with significant differences between the two groups: ASA classification at resection of CRLM, emergence time of CRLM, distribution of CRLM, number of CRLMs, preoperative adjuvant chemotherapy for CRLM, and postoperative adjuvant chemotherapy for CRLM. Except for postoperative adjuvant chemotherapy, all were independent prognostic factors in groups Y and/or E. The median PS was 0.05 (0.01–0.29) in group Y and 0.18 (0.01–0.29) in group E (*p* < 0.0001). The c‐statistic of 0.73 (95% CI: 0.70–0.77, *p* < 0.0001) showed satisfactory discrimination. A total of 212 patients in each group were matched in one‐on‐one pair PSM analysis. The standardized differences indicated that the two groups were well‐balanced for the factors with significant differences before PSM, and there were no significant differences in univariate analysis of clinicopathological factors between the groups after PSM (Table S1).

### 3.6 RFS, CSS and OS in the Propensity‐Matched Cohort

In the PSM cohort, group E showed a comparable RFS to that in group Y (5‐year RFS; group Y: 40.9%, group E: 33.8%) (HR: 1.20, 95% confidence interval (CI): 0.93–1.54, *p* = 0.16) (Figure 1d), and had significantly worse CSS (5‐year CSS: group Y: 70.5%, group E: 53.4%) (HR: 1.77, 95% CI: 1.24–2.53, *p* = 0.002) (Figure 1e) and OS (5‐year OS: group Y: 66.5%, group E: 47.3%) (HR: 1.80, 95% CI: 1.30–2.49, *p* = 0.0004) (Figure 1f).

### 3.7 Treatment for Recurrence After Initial Liver Resection for CRLM

**FIGURE 2:**
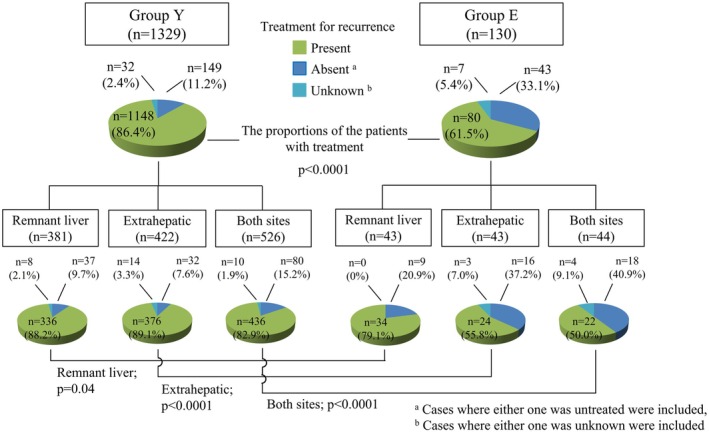
Treatment for recurrence after initial liver resection for CRLM There were 1329 patients with recurrence in group Y and 130 patients with recurrence in group E. Treatment for recurrence was given at a significantly lower rate in group E (80/130 (61.5%) vs. 1148/1329 (86.4%), *p* < 0.001).

There were 1329 patients with recurrence in group Y and 130 patients with recurrence in group E (Figure 2). Treatment for recurrence was given at a significantly lower rate in group E (80/130 (61.5%) vs. 1148/1329 (86.4%), *p* < 0.001). The recurrence pattern was stratified into the remnant liver, extrahepatic, and both remnant liver and extrahepatic. Around 80%–90% of patients in group Y received treatment for each recurrence pattern (Figure 2), whereas the rates of treatment in group E were significantly lower: only 79.1% for the remnant liver (*p* = 0.04), 55.8% for extrahepatic recurrence (*p* < 0.001), and 50.0% for recurrence at both sites (*p* < 0.001). The details of treatment for recurrence after initial resection of CRLM in groups Y and E are shown in Table 4. The proportion of patients in group E who underwent surgical resection alone or combined with chemotherapy or received chemotherapy alone for any type of recurrence was lower than that in group Y.

**TABLE 4:** Details of treatment for recurrence after initial resection of CRLM in groups Y and E.

Treatment		Group Y (*n* = 1329)				Group E (*n* = 130)			
		Remnant liver *n* = 381 (%)	Extrahepatic *n* = 422 (%)	Both sites *n* = 526 (%)		Remnant liver *n* = 43 (%)	Extrahepatic *n* = 43 (%)	Both sites *n* = 44 (%)	
				Remnant liver	Extrahepatic			Remnant liver	Extrahepatic
Surgical resection (+)	Surgical resection alone	143 (37.5%)	95 (22.5%)	89 (16.9%)	53 (10.1%)	13 (30.2%)	7 (16.3%)	4 (9.1%)	4 (9.1%)
	Surgical resection + chemotherapy	57 (15.0%)	39 (9.2%)	65 (12.4%)	39 (7.4%)	2 (4.7%)	0 (0%)	3 (6.8%)	2 (4.5%)
	Surgical resection + radiation	1 (0.3%)	1 (0.2%)	1 (0.2%)	0 (0%)	0 (0%)	0 (0%)	0 (0%)	1 (2.3%)
	Surgical resection + chemotherapy + radiation	0 (0%)	5 (1.2%)	1 (0.2%)	3 (0.6%)	0 (0%)	0 (0%)	0 (0%)	1 (2.3%)
	Surgical resection + RFA/MCT	1 (0.3%)	1 (0.2%)	0 (0%)	0 (0%)	0 (0%)	0 (0%)	0 (0%)	0 (0%)
	Surgical resection + chemotherapy + RFA/MCT	3 (0.8%)	0 (0%)	2 (0.4%)	0 (0%)	0 (0%)	0 (0%)	0 (0%)	0 (0%)
Surgical resection (−)	Chemotherapy alone	118 (31.0%)	206 (48.8%)	265 (50.4%)	323 (61.4%)	13 (30.2%)	17 (39.5%)	13 (29.5%)	13 (29.5%)
	Chemotherapy + radiation	0 (0%)	16 (3.8%)	5 (1.0%)	7 (1.3%)	0 (0%)	0 (0%)	0 (0%)	0 (0%)
	Chemotherapy + RFA/MCT	2 (0.5%)	1 (0.2%)	1 (0.2%)	0 (0%)	3 (7.0%)	0 (0%)	0 (0%)	0 (0%)
	Radiation alone	4 (1.0%)	12 (2.8%)	2 (0.4%)	9 (1.7%)	1 (2.3%)	0 (0%)	0 (0%)	1 (2.3%)
	RFA/MCT alone	7 (1.8%)	0 (0%)	5 (1.0%)	2 (0.4%)	2 (4.7%)	0 (0%)	2 (4.5%)	0 (0%)
	None^a^	37 (9.7%)	32 (7.6%)	80 (15.2%)^a^		9 (20.9%)	16 (37.2%)	18 (40.9%)^a^	
	Unknown^b^	8 (2.1%)	14 (3.3%)	10 (1.9%)^b^		0 (0%)	3 (7.0%)	4 (9.1%)^b^	

^a^ Cases where either one was untreated were included.

^b^ Cases where either one was unknown were included.

### 3.8 Clinicopathological Factors Related to the Absence of Treatment for Recurrence After Initial Liver Resection for CRLM

**FIGURE 3:**
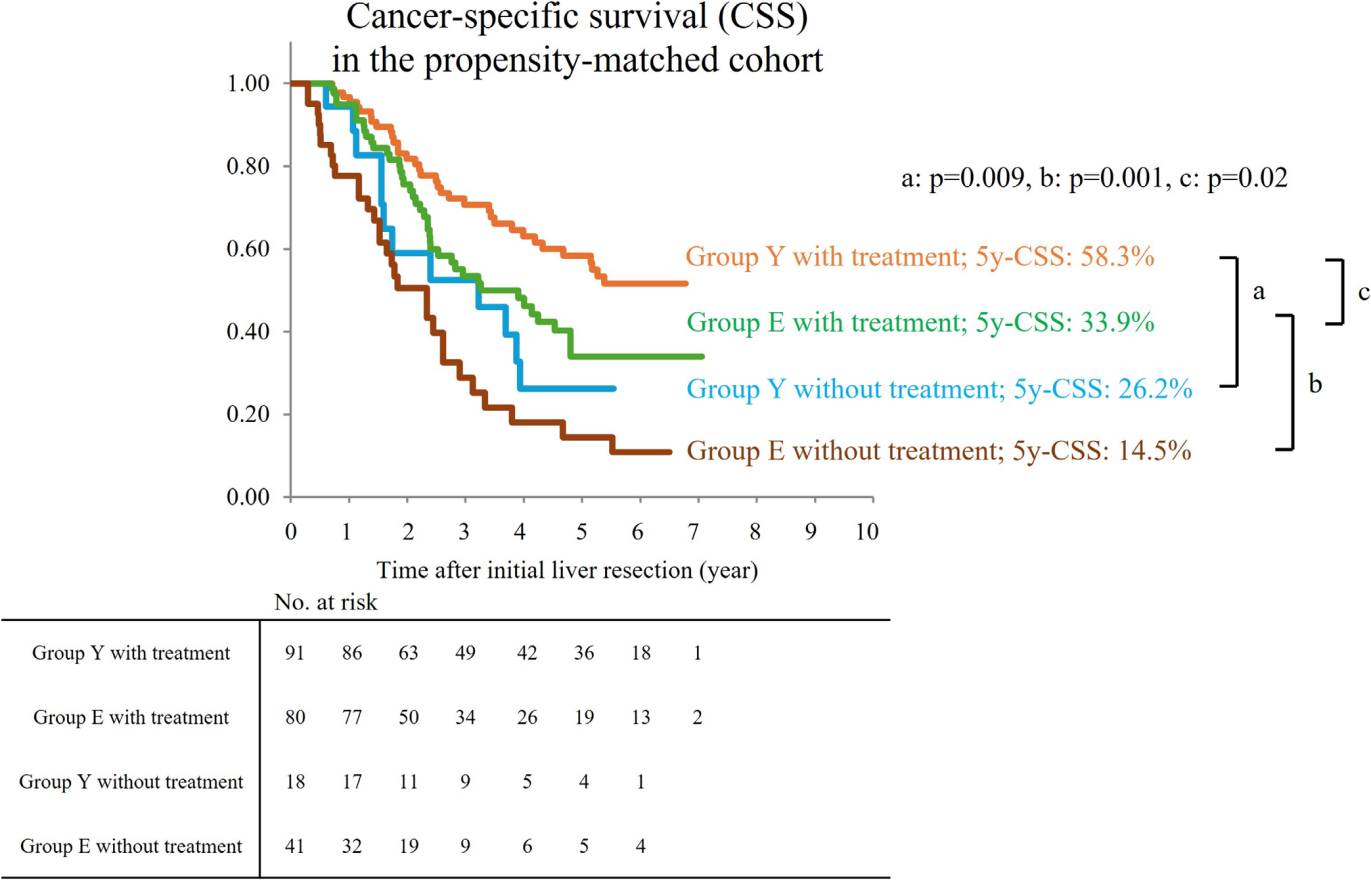
CSS in the propensity‐matched cohort by age group and presence/absence of treatment for recurrence Patients in both groups Y and E with treatment had significantly worse CSS compared to those without treatment (*p* = 0.009 and 0.001, respectively). In addition, group E with treatment had significantly worse CSS compared to group Y with treatment (*p* = 0.02).

In univariate analysis for clinicopathological factors related to the absence of treatment for recurrence after initial liver resection for CRLM in group Y, patients without this treatment more frequently had an ASA classification at resection of CRLM ≥ 3 (*p* = 0.03) and anatomical liver resection (*p* < 0.0001) (Table S2). Multivariate analysis revealed that ASA classification at resection of CRLM ≥ 3 (OR: 2.28, 95% CI: 1.12–4.63, *p* = 0.02) and anatomical liver resection (OR: 2.68, 95% CI: 1.85–3.87, *p* < 0.0001) were independent significant factors related to the absence of treatment for recurrence. Similarly, in univariate analysis in group E, patients without treatment for recurrence more frequently had an ASA classification at resection of CRLM ≥ 3 (*p* = 0.03) and synchronous liver metastasis (*p* = 0.01), and less frequently had postoperative adjuvant chemotherapy for CRLM (*p* = 0.002) (Table S3). Multivariate analysis revealed that synchronous liver metastasis (OR: 3.00, 95% CI: 1.33–7.02, *p* = 0.008) and the absence of postoperative adjuvant chemotherapy for CRLM (OR: 5.22, 95% CI: 1.78–19.37, *p* = 0.002) were independent significant factors related to the absence of treatment for recurrence. CSS in the propensity‐matched cohort was compared by age group and presence/absence of treatment for recurrence (Figure 3). Patients in both groups Y and E with treatment had significantly worse CSS compared to those without treatment (*p* = 0.009 and 0.001, respectively). In addition, group E with treatment had significantly worse CSS compared to group Y with treatment (*p* = 0.02).

## 4 Discussion

Over the past two to three decades, surgical resection of CRLM has evolved into a standard treatment [16, 19]. In the current study, a recently treated large cohort was used to investigate the significance of surgical resection of CRLM in elderly patients aged ≥ 80. RFS was found to be comparable between patients aged < 80 and ≥ 80, but CSS and OS were significantly worse in those aged ≥ 80, even after PSM. The significantly worse CSS and OS may have resulted from differences in rates of treatment for recurrence between the two patient populations. To the best of our knowledge, this is the largest cohort study of elderly patients aged ≥ 80 with CRLM. A previous study showed a moderate correlation between RFS and OS in surgically treated patients with CRLM [32]. However, the study also revealed a negative impact of age (≥ 75 vs. < 75) on survival after initial liver resection for CRLM.

As expected, there were significant differences in patient backgrounds between the two groups, with more elderly patients aged ≥ 80 with fewer tumors selected for surgical resection for CRLM. Previous reports comparing octogenarians with patients aged 70–79 years have also found that liver‐metastatic burden is less aggressive in octogenarians [18, 22]. In our study, elderly patients aged ≥ 80 did not have significantly worse RFS, even after balancing adjustment factors using PSM. Therefore, to some extent, surgical resection seems to be as effective in elderly patients aged ≥ 80, excluding high tumor‐bearing cases, as in nonelderly patients. Meta‐analyses of large cohorts have found similar disease‐free survival in patients with CRLM aged ≥ 70 and < 70 [21, 33], supporting the survival benefit of liver resection in selected elderly patients (≥ 70 in these analyses). However, a prospective direct comparison in selected octogenarians with and without surgical resection is needed to reach a definitive conclusion.

Interestingly, in the current study, ASA classification ≥ 3 at resection of CRLM was not found to be a prognostic factor for RFS, CSS and OS in group E, but was an independent prognostic factor for OS in group Y. The proportions of Japanese patients with ASA classification ≥ 3 who underwent curative resection for Stage I‐III CRC have been reported to be 24.5% for age ≥ 85 (*n* = 49), 14.6% for age 75–84 (*n* = 673) and 4.3% for age < 70 (*n* = 1144) in a similar period (2016–2020) [34]. In the current study, the rate of patients with ASA classification ≥ 3 in group E (7.9%) was about half of that for the 75–84 age group in the previous study, while that in group Y (3.7%) was similar to that for the < 70 age group. Therefore, many patients aged ≥ 80 with CRLM with ASA classification ≥ 3 may have been excluded from surgical resection of CRLM. This selection bias could have led to the finding that ASA classification ≥ 3 at resection of CRLM was not a prognostic factor for RFS, CSS and OS in group E.

Previous reports have shown no significant difference in CSS or OS between patients aged 70–79 and octogenarians [18, 22]. However, these reports included less than 100 patients aged ≥ 80. A meta‐analysis showing significantly worse OS in patients aged ≥ 70 suggested that most elderly patients may not be treated with chemotherapy [33]. A population‐based study also revealed inferior CSS and OS in patients aged ≥ 75 after adjustment for patient backgrounds [16]. A previous study in a large Japanese cohort also revealed that age (> 70) was an independent predictive factor for OS, but not for DFS [35]. The current study using real‐world data revealed a clear trend for elderly patients aged ≥ 80 to be less likely to receive treatment for recurrence, especially for extrahepatic lesions. A previous report also showed lower rates of treatment for recurrence, including surgical resection or chemotherapy or both, for elderly patients aged ≥ 80 [18]. In addition, elderly patients aged ≥ 80 with synchronous CRLM and/or without postoperative adjuvant chemotherapy for CRLM tended not to receive treatment for recurrence. Such elderly patients are thought not to be willing to receive treatment for recurrence, unlike elderly patients aged ≥ 80 with metachronous CRLM and/or postoperative adjuvant chemotherapy for CRLM. In addition, the worse CSS in group E with treatment compared to group Y with treatment may have been due to differences in treatment, including regimens or cycles of chemotherapy for recurrence. However, such detailed information was not included in the database. It has been reported that development of chemotherapy‐associated liver injury following oxaliplatin‐based chemotherapy in CRLM does not differ among age groups of < 65, 65–74 and > 75 years [36]. However, physicians should keep this finding in mind with regard to limitations of treatments for recurrence after initial liver resection, especially for elderly patients aged ≥ 80 with prognostic factors related to RFS, CSS and OS, as shown in Table 3.

There are some limitations in this study. First, the study was retrospective. Second, we could not examine the effectiveness of surgical resection for CRLM because long‐term outcomes such as CSS or OS were not compared between elderly patients with and without surgical treatment. In the retrospective data, the reasons why surgical resection was not chosen were unclear because data on general condition, such as ASA classification, were lacking in patients without surgical treatment. Third, the study was limited to liver metastatic disease, and the results may not be applicable to patients with extrahepatic metastasis. However, based on our results, an elderly patient aged ≥ 80 with extrahepatic metastases at the first visit may not be thought of as a candidate for aggressive treatment, including surgical resection. Fourth, the regimens of pre‐ and postoperative adjuvant chemotherapy were not consistent among institutions, since the indication and regimen for each case was decided in a discussion between the physician and patient. Therefore, various regimens may have been used, including molecular‐targeted agents. We also had no data on the details of the second or later line treatment for recurrence after the initial surgical resection. The surgical tolerance also provides important information. However, the detailed reason for the decision to not use repeat resection for recurrence in the remnant liver or chemotherapy for the extrahepatic recurrence in (physical status, psychological status, oncological status or a combination) was not available in the database. Finally, our findings are based on Japanese data [35] and require validation using data from the US or Europe.

In conclusion, this study provides important insights into management and outcomes of elderly patients aged ≥ 80 with CRLM. RFS was comparable between patients aged < 80 and ≥ 80. Therefore, surgical resection for CRLM can be as effective in carefully selected elderly patients aged ≥ 80 as in nonelderly patients, provided that clinicians consider the identified limitations regarding treatment intensity for recurrence. However, CSS and OS were significantly worse in elderly patients aged ≥ 80, even after PSM. These outcomes may be due to a lower rate of treatment for recurrence in patients aged ≥ 80. Physicians should keep this finding in mind with regard to limitations of treatments for recurrence after initial liver resection in elderly patients aged ≥ 80 with CRLM.

## Author Contributions

**Kiichi Sugimoto:** conceptualization, methodology, data curation, formal analysis, writing – original draft. **Akio Saiura:** writing – review and editing, methodology, supervision. **Shuko Nojiri:** writing – review and editing, methodology. **Hirotoshi Kobayashi:** writing – review and editing, supervision. **Goro Honda:** writing – review and editing, supervision. **Kazushige Kawai:** supervision, writing – review and editing. **Kiyoshi Hasegawa:** supervision, writing – review and editing. **Keiichi Takahashi:** supervision, writing – review and editing, methodology.

## Funding

The authors have nothing to report.

## Ethics Statement

This study was performed in accordance with the ethical standards of the 1964 Declaration of Helsinki and its later amendments.

## Conflicts of Interest

Akio Saiura is a member of the Editorial Board.

## Supporting information


**Figure S1:** The scheme of the study.

**Table S1:** Clinicopathological factors in the groups Y and E after propensity score matching.

**Table S2:** Clinicopathological factors related to the absence of treatment for recurrence after initial liver resection for CRLM in group Y.

**Table S3:** Clinicopathological factors related to the absence of treatment for recurrence after initial liver resection for CRLM in group E.
