# Supplementary figures and images for: Surgical Resection for Colorectal Liver Metastasis in Elderly Patients Aged ≥ 80: A Retrospective Nationwide Cohort Survey in Japan With Propensity Score Matching
Source: Ann Gastroenterol Surg. 2026 Mar 10;10(4):1107–19. doi: 10.1002/ags3.70213 (PMC13326831; doi:10.1002/ags3.70213)

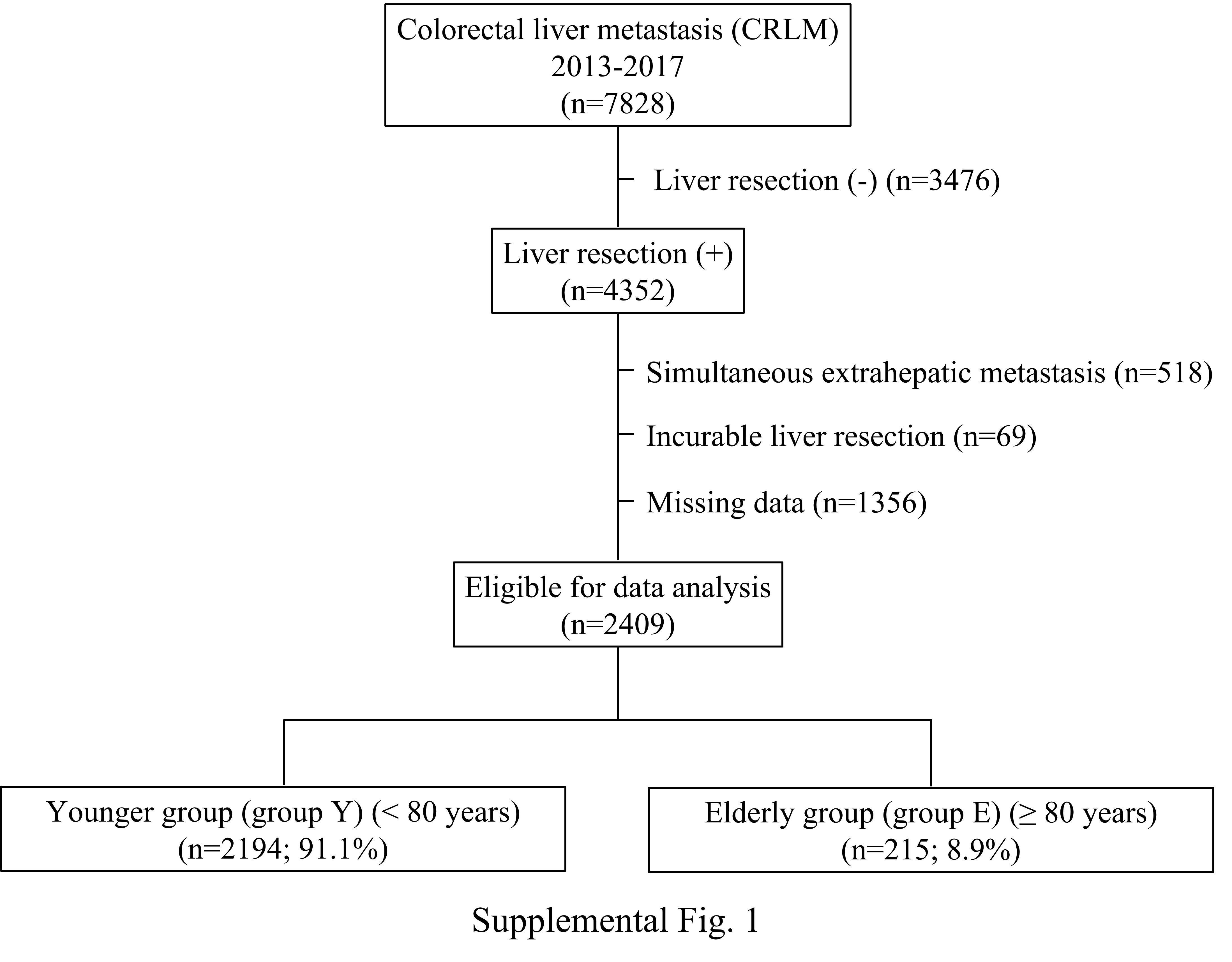

Supplement: Supplementary file 1 — Figure S1: The scheme of the study. [file AGS3-10-1107-s001.jpg]
