# Supplementary material for: Surgical Resection for Colorectal Liver Metastasis in Elderly Patients Aged ≥ 80: A Retrospective Nationwide Cohort Survey in Japan With Propensity Score Matching
Source: Ann Gastroenterol Surg. 2026 Mar 10;10(4):1107–19. doi: 10.1002/ags3.70213 (PMC13326831; doi:10.1002/ags3.70213)
Supplement: Supplementary file 2 — Table S1: Clinicopathological factors in the groups Y and E after propensity score matching. [file AGS3-10-1107-s004.doc]

| Supplemental Table 1 Clinicopathological factors in the groups Y and E after propensity score matching | | | | | |
| --- | --- | --- | --- | --- | --- |
| Clinicopathological factors | Variables | After matching | | p-value | Standardized  difference |
|  |  | Group Y  n=212 (%) | Group E  n=212 (%) |  |  |
| Gender | Male  Female | 141 (66.5%)  71 (33.5%) | 123 (58.0%)  89 (42.0%) | 0.09 | 0.18 |
| ASA classification ^a)^  at resection of CRLM | ≤ 2  ≥ 3 | 198 (93.4%)  14 (6.6%) | 198 (93.4%)  14 (6.6%) | 1.00 | 0.00 |
| Locations of  primary tumor ^b)^ | Colon  Rectum | 158 (74.5%)  54 (25.5%) | 170 (80.2%)  42 (19.8%) | 0.20 | 0.14 |
| Undifferentiated component  in primary tumor | Absent  Present | 189 (89.2%)  23 (10.9%) | 199 (93.9%)  13 (6.1%) | 0.12 | 0.29 |
| Depth of tumor invasion | pT/ypT 1-3  pT/ypT 4 | 158 (74.5%)  54 (25.5%) | 151 (71.2%)  61 (28.8%) | 0.51 | 0.07 |
| Lymph node metastasis | pN/ypN 0  pN/ypN 1, 2 | 79 (37.3%)  133 (62.7%) | 89 (42.0%)  123 (58.0%) | 0.37 | 0.10 |
| Emergence time  of CRLM | Synchronous  Metachronous | 83 (39.2%)  129 (60.9%) | 83 (39.2%)  129 (60.9%) | 1.00 | 0.00 |
| Distribution  of CRLM | Unilobar  Bilobar | 179 (84.4%)  33 (15.6%) | 179 (84.4%)  33 (15.6%) | 1.00 | 0.00 |
| Number of CRLM | ≤ 3  ≥ 4 | 192 (90.6%)  20 (9.4%) | 192 (90.6%)  20 (9.4%) | 1.00 | 0.00 |
| Maximum diameter  of CRLM | < 50mm  ≥ 50mm | 188 (88.7%)  24 (11.3%) | 185 (87.3%)  27 (12.7%) | 0.77 | 0.04 |
| Liver resection procedure ^c)^ | Anatomical  Partial | 72 (36.6%)  125 (63.5%) | 84 (42.4%)  114 (57.6%) | 0.26 | 0.12 |
| Surgical curability  of CRLM | R0  R1 | 202 (95.3%)  10 (4.7%) | 197 (92.9%)  15 (7.1%) | 0.41 | 0.10 |
| Postoperative complication 　　　　　　　　　　after liver resection ≥ Grade 3 ^d)^ | Absent  Present | 186 (91.6%)  17 (8.4%) | 178 (89.0%)  22 (11.0%) | 0.40 | 0.45 |
| Preoperative adjuvant chemotherapy for CRLM | Absent  Present | 190 (89.6%)  22 (10.4%) | 190 (89.6%)  22 (10.4%) | 1.00 | 0.00 |
| Postoperative adjuvant chemotherapy for CRLM | Absent  Present | 170 (80.2%)  42 (19.8%) | 170 (80.2%)  42 (19.8%) | 1.00 | 0.00 |

^a)^ American Society of Anesthesiologists classification.

^b)^ Multiple cancers were included. Cases involving rectal lesions were classified as 'rectum'.

^c)^ Data were missing in 29 patients.

^d)^ Clavien-Dindo classification, data were missing in 21 patients.
