# Supplementary material for: Surgical Resection for Colorectal Liver Metastasis in Elderly Patients Aged ≥ 80: A Retrospective Nationwide Cohort Survey in Japan With Propensity Score Matching
Source: Ann Gastroenterol Surg. 2026 Mar 10;10(4):1107–19. doi: 10.1002/ags3.70213 (PMC13326831; doi:10.1002/ags3.70213)
Supplement: Supplementary file 3 — Table S2: Clinicopathological factors related to the absence of treatment for recurrence after initial liver resection for CRLM in group Y. [file AGS3-10-1107-s002.doc]

| Supplemental Table 2 Clinicopathological factors related to the absence of treatment for recurrence after initial liver resection for CRLM in group Y | | | | | | | | |
| --- | --- | --- | --- | --- | --- | --- | --- | --- |
| Clinicopathological factors | Variables | Treatment for recurrence | |  | Univariate |  | Multivariate | |
|  |  | Present  n=1148 (%) | Absent  n=149 (%) |  | p-  value |  | OR  (95%CI) | p-value |
| Gender | Male  Female | 729 (63.5%)  419 (36.5%) | 89 (59.7%)  60 (40.3%) |  | 0.37 |  |  |  |
| ASA classification ^a)^  at resection of CRLM | ≤ 2  ≥ 3 | 1106 (96.3%)  42 (3.7%) | 137 (92.0%)  12 (8.1%) |  | 0.03 |  | 2.28  (1.12-4.63) | 0.02 |
| Locations of  primary tumor ^b)^ | Colon  Rectum | 858 (74.7%)  290 (25.3%) | 116 (77.9%)  33 (22.2%) |  | 0.48 |  |  |  |
| Undifferentiated component  in primary tumor | Absent  Present | 1047 (91.2%)  101 (8.8%) | 132 (88.6%)  17 (11.4%) |  | 0.29 |  |  |  |
| Depth of tumor invasion | pT/ypT 1-3  pT/ypT 4 | 758 (66.0%)  390 (34.0%) | 110 (73.8%)  39 (26.2%) |  | 0.06 |  |  |  |
| Lymph node metastasis | pN/ypN 0  pN/ypN 1, 2 | 346 (30.1%)  802 (69.9%) | 42 (28.2%)  107 (71.8%) |  | 0.70 |  |  |  |
| Emergence time  of CRLM | Synchronous  Metachronous | 717 (62.5%)  431 (37.5%) | 102 (68.5%)  47 (31.5%) |  | 0.18 |  |  |  |
| Distribution  of CRLM | Unilobar  Bilobar | 663 (57.8%)  485 (42.3%) | 93 (62.4%)  56 (37.6%) |  | 0.29 |  |  |  |
| Number of CRLM | ≤ 3  ≥ 4 | 826 (72.0%)  322 (28.1%) | 112 (75.2%)  37 (24.8%) |  | 0.44 |  |  |  |
| Maximum diameter  of CRLM | < 50mm  ≥ 50mm | 932 (81.2%)  216 (18.8%) | 122 (81.9%)  27 (18.1%) |  | 0.91 |  |  |  |
| Liver resection procedure ^c)^ | Anatomical  Partial | 393 (38.3%)  632 (61.7%) | 86 (62.3%)  52 (37.7%) |  | < 0.0001 |  | 2.68  (1.85-3.87) | < 0.0001 |
| Surgical curability  of CRLM | R0  R1 | 1074 (93.6%)  74 (6.5%) | 145 (97.3%)  4 (2.7%) |  | 0.10 |  |  |  |
| Postoperative complication 　　　　　　　　　　after liver resection ≥ Grade 3 ^d)^ | Absent  Present | 977 (90.4%)  104 (9.6%) | 100 (93.5%)  7 (6.5%) |  | 0.38 |  |  |  |
| Preoperative adjuvant chemotherapy for CRLM | Absent  Present | 877 (76.4%)  271 (23.6%) | 119 (79.9%)  30 (20.1%) |  | 0.41 |  |  |  |
| Postoperative adjuvant chemotherapy for CRLM | Absent  Present | 493 (42.9%)  655 (57.1%) | 74 (49.7%)  75 (50.3%) |  | 0.14 |  |  |  |

^a)^ American Society of Anesthesiologists classification

^b)^ Multiple cancers were included. Cases involving rectal lesions were classified as 'rectum'.

^c)^ Data were missing in 134 patients

^d)^ Clavien-Dindo classification, data were missing in 109 patients
