# Supplementary material for: Surgical Resection for Colorectal Liver Metastasis in Elderly Patients Aged ≥ 80: A Retrospective Nationwide Cohort Survey in Japan With Propensity Score Matching
Source: Ann Gastroenterol Surg. 2026 Mar 10;10(4):1107–19. doi: 10.1002/ags3.70213 (PMC13326831; doi:10.1002/ags3.70213)
Supplement: Supplementary file 4 — Table S3: Clinicopathological factors related to the absence of treatment for recurrence after initial liver resection for CRLM in group E. [file AGS3-10-1107-s003.doc]

| Supplemental Table 3 Clinicopathological factors related to the absence of treatment for recurrence after initial liver resection for CRLM in group E | | | | | | | | |
| --- | --- | --- | --- | --- | --- | --- | --- | --- |
| Clinicopathological factors | Variables | Treatment for recurrence | |  | Univariate |  | Multivariate | |
|  |  | Present  n=80 (%) | Absent  n=43 (%) |  | p-  value |  | OR  (95%CI) | p-value |
| Gender | Male  Female | 45 (56.3%)  35 (43.8%) | 25 (58.1%)  18 (41.9%) |  | 0.85 |  |  |  |
| ASA classification ^a)^  at resection of CRLM | ≤ 2  ≥ 3 | 77 (96.3%)  3 (3.8%) | 36 (83.7%)  7 (16.3%) |  | 0.03 |  | 3.03  (0.74-15.43) | 0.12 |
| Locations of  primary tumor ^b)^ | Colon  Rectum | 65 (81.3%)  15 (18.8%) | 36 (83.7%)  7 (16.3%) |  | 0.81 |  |  |  |
| Undifferentiated component  in primary tumor | Absent  Present | 78 (97.5%)  2 (2.5%) | 41 (95.4%)  2 (4.7%) |  | 0.61 |  |  |  |
| Depth of tumor invasion | pT/ypT 1-3  pT/ypT 4 | 54 (67.5%)  26 (32.5%) | 26 (60.5%)  17 (39.5%) |  | 0.44 |  |  |  |
| Lymph node metastasis | pN/ypN 0  pN/ypN 1, 2 | 25 (31.3%)  55 (68.8%) | 13 (30.2%)  30 (69.8%) |  | 1.00 |  |  |  |
| Emergence time  of CRLM | Synchronous  Metachronous | 34 (42.5%)  46 (57.5%) | 29 (67.4%)  14 (32.6%) |  | 0.01 |  | 3.00  (1.33-7.02) | 0.008 |
| Distribution  of CRLM | Unilobar  Bilobar | 62 (77.5%)  18 (22.5%) | 33 (76.7%)  10 (23.3%) |  | 1.00 |  |  |  |
| Number of CRLM | ≤ 3  ≥ 4 | 69 (86.3%)  11 (13.8%) | 37 (86.1%)  5 (14.0%) |  | 1.00 |  |  |  |
| Maximum diameter  of CRLM | < 50mm  ≥ 50mm | 68 (85.0%)  12 (15.0%) | 33 (76.7%)  10 (23.3%) |  | 0.32 |  |  |  |
| Liver resection procedure ^c)^ | Anatomical  Partial | 30 (40.5%)  44 (59.5%) | 16 (40.0%)  24 (60.0%) |  | 1.00 |  |  |  |
| Surgical curability  of CRLM | R0  R1 | 75 (93.8%)  5 (6.3%) | 37 (86.1%)  6 (14.0%) |  | 0.19 |  |  |  |
| Postoperative complication 　　　　　　　　　　after liver resection ≥ Grade 3 ^d)^ | Absent  Present | 7 (9.1.%)  70 (90.9%) | 6 (15.0%)  34 (85.0%) |  | 0.36 |  |  |  |
| Preoperative adjuvant chemotherapy for CRLM | Absent  Present | 70 (87.5%)  10 (12.5%) | 38 (88.4%)  5 (11.6%) |  | 1.00 |  |  |  |
| Postoperative adjuvant chemotherapy for CRLM | Absent  Present | 52 (65.0%)  28 (35.0%) | 39 (90.7%)  4 (9.3%) |  | 0.002 |  | 5.22  (1.78-19.37) | 0.002 |

^a)^ American Society of Anesthesiologists classification

^b)^ Multiple cancers were included. Cases involving rectal lesions were classified as 'rectum'.

^c)^ Data were missing in 9 patients

^d)^ Clavien-Dindo classification, data were missing in 6 patients
